# Beyond the Guidelines: The Grey Zones of the Management of Gastric Cancer. Consensus Statements from the Gastric Cancer Italian Network (GAIN)

**DOI:** 10.3390/cancers13061304

**Published:** 2021-03-15

**Authors:** Lorenzo Fornaro, Andrea Spallanzani, Ferdinando de Vita, Domenico D’Ugo, Alfredo Falcone, Laura Lorenzon, Giuseppe Tirino, Stefano Cascinu, on behalf of GAIN (GAstric Cancer Italian Network)

**Affiliations:** 1 Department of Translational Medicine, Division of Medical Oncology, AOU Pisana, 56126 Pisa, Italy; lorenzo.fornaro@gmail.com; 2 Department of Oncology and Hematology, University Hospital of Modena, 41125 Modena, Italy; andrea.spallanzani@gmail.com; 3 Department of Precision Medicine, Division of Medical Oncology, School of Medicine, University of Campania ‘Luigi Vanvitelli’, 81100 Caserta, Italy; ferdinando.devita@unicampania.it (F.d.V.); giuseppe.tirino@unicampania.it (G.T.); 4 General Surgery Unit, Fondazione Policlinico Universitario Agostino Gemelli IRCCS, Catholic University, 00168 Rome, Italy; domenicodugo@icloud.com (D.D.); laura.lorenzon@policlinicogemelli.it (L.L.); 5 Department of Translational Medicine, Division of Medical Oncology, University of Pisa, 56126 Pisa, Italy; alfredo.falcone@med.unipi.it; 6 Medical Oncology, Università Vita-Salute San Raffaele, 20132 Milan, Italy

**Keywords:** gastric cancer, GEJ adenocarcinoma, recommendations, treatment, diagnosis

## Abstract

**Simple Summary:**

Management of gastric and gastroesophageal junction (GEJ) adenocarcinoma remains challenging, because of the heterogeneity in tumor biology within the upper gastrointestinal tract. This manuscript is the product of a formal consensus process conducted in three Delphi rounds and a consensus meeting by the GAIN (GAstric cancer Italian Network) group. The goal of this document is to present a synthesis of available evidence and, where this is lacking, to provide expert opinion directed at prevention, diagnosis, and proper management of gastric and GEJ adenocarcinoma, and in particular aspects of practical management not fully supported by guidelines.

**Abstract:**

Background: Management of gastric and gastroesophageal junction (GEJ) adenocarcinoma remains challenging, because of the heterogeneity in tumor biology within the upper gastrointestinal tract. Daily clinical practice is full of grey areas regarding the complexity of diagnostic, staging, and therapeutic procedures. The aim of this paper is to provide a guide for clinicians facing challenging situations in routine practice, taking a multidisciplinary consensus approach based on available literature. Methods: The GAIN (GAstric cancer Italian Network) group was established with the aims of reviewing literature evidence, discussing key issues in prevention, diagnosis, and management of gastric and GEJ adenocarcinoma, and offering a summary of statements. A Delphi consensus method was used to obtain opinions from the expert panel of specialists. Results: Forty-nine clinical questions were identified in six areas of interest: role of multidisciplinary team; risk factors; diagnosis; management of early gastric cancer and multimodal approach to localized gastric cancer; treatment of elderly patients with locally advanced resectable disease; and treatment of locally advanced and metastatic cancer. Conclusions: The statements presented may guide clinicians in practical management of this disease.

## 1. Introduction

Gastric cancer represents a significant health problem—it is the third leading cause of cancer death worldwide, although incidence varies widely [1]. Management of gastric and gastroesophageal junction (GEJ) adenocarcinoma remains challenging, because of the heterogeneity of tumor biology within the upper gastrointestinal tract; the complexity of diagnostic, staging, and therapeutic procedures; and differences in treatment algorithms [2]. As a consequence, a multidisciplinary approach has long been advocated as crucial to personalize management [3,4].

Although several authoritative guidelines are available, daily practice is full of uncertainties, as high-quality evidence and conclusive studies are lacking. The GAIN (GAstric cancer Italian Network) group was established with the aim of filling this gap between evidence and practice. Experts in the field selected the major open questions in the management of gastric and GEJ adenocarcinoma, reviewed literature evidence, discussed key issues in prevention, diagnosis, and management, and provided recommendations to be used in clinical practice.

## 2. Methods

This multidisciplinary consensus was developed by the GAIN group, comprising 19 oncologists, five surgical oncologists, one gastroenterologist, one pathologist, one nuclear doctor, one radiologist, one radiation oncologist, one nutritionist, and a patient representative. Clinical centers were chosen according to their expertise in gastric cancer management across Italy.

A Delphi consensus method was used to obtain opinions from the panel. Two preparatory meetings, three Delphi rounds, and a final consensus conference took place between May 2019 and March 2020. In the preparatory meetings, decisions were made on the final scope and structure of the project, composition of the expert panel, methods, and topics, including relevant clinical questions that the consensus should address. Forty-nine clinical questions were identified in six areas of interest: role of multidisciplinary team; risk factors; diagnosis; management of early gastric cancer and multimodal approach to localized gastric cancer; treatment of elderly patients with locally advanced resectable disease; treatment of locally advanced and metastatic cancer.

A group of experts (three oncologists and two surgical oncologists) reviewed the literature to answer the clinical questions identified and proposed a draft of statements with supporting evidence for each topic. Key issues were discussed at a second face-to-face meeting in July 2019, after which a comprehensive document was circulated to the expanded group of experts. Each expert was asked to comment and suggest modifications to the draft statements through a Delphi method. Throughout Delphi rounds, participants were asked to rate their agreement with each statement with a three-point Likert scale: disagree, abstain, agree; consensus was defined as >70% “agree” with <15% “disagree”. Statements with levels of agreement <70% were re-worded or were clarified with additional words in subsequent Delphi rounds, in response to respondent’s comments. If no final agreement was reached after the consensus conference, those statements were eliminated (statements included: 74 out of 82 initial statements). These suggestions were made available to the other experts in a series of web-based discussion rounds (three online rounds) for further discussion and definitive approval. A final consensus online meeting was held on 26 March 2020.

## 3. Results

### 3.1. Role of Multidisciplinary Team

**Unanswered questions**: What is the impact of multidisciplinary discussion on therapeutic decision-making in patients with gastroesophageal cancer? Which specialists should be involved in the multidisciplinary team (MDT)?

#### 3.1.1. Statements

All newly diagnosed gastroesophageal cancer cases must be discussed within the MDT and regularly reviewed as this is associated with improvements in overall management (from diagnosis to staging and treatment). The MDT should comprise an abdominal surgeon, radiation oncologist, medical oncologist, pathologist, radiologist, gastroenterologist, and a specialist in clinical nutrition.

#### 3.1.2. Sources of Evidence

Management of gastroesophageal cancers requires complex clinical decision-making. MDT involvement is required to ensure timely and appropriate care by different specialists. Several studies confirm that roundtable discussions within MDTs are associated with improvements in staging/diagnosis, survival improvement, adherence to international guidelines, and promoting the execution of multimodal treatments in many cases [5,6,7,8].

Usually, patients with gastroesophageal cancer have weight loss at diagnosis—an indicator of malnutrition or risk for developing malnutrition. Malnutrition and sarcopenia (particularly reduced lean body mass) have prognostic bearing on outcomes at all disease stages [9,10]. Involvement of dedicated nutritional specialists increases the chance of successful outcome. The nutrition team should include at least one physician (internist, gastroenterologist, endocrinologist, or nutritionist) and a dietitian [11,12].

### 3.2. Risk Factors

#### 3.2.1. Precancerous Conditions: Chronic Gastritis with Intestinal-Type Metaplasia, Chronic Atrophic Gastritis

**Unanswered questions**: How are higher-risk populations defined? Which diagnostic tools and staging procedures can be recommended in suspected precancerous conditions? How is appropriate follow-up of precancerous conditions chosen?

##### Statements

(1) An esophagogastroduodenoscopy (EGDS) should be performed on all patients presenting with new onset dyspeptic symptoms, regardless of the presence of gastroesophageal reflux or other risk factors such as active smoking or *H. pylori* positivity. Population screening is recommended in Asian countries but there is no evidence to support it in Western populations.
(2) Early identification of patients with chronic atrophic gastritis and intestinal metaplasia (including Barrett) is fundamental to endoscopic staging by the updated Sydney system, with an accurate assessment of the degree of atrophy/metaplasia with OLGA (Operative Link on Gastritis Assessment) and OLGIM (Operative Link on Gastritis/Intestinal-Metaplasia Assessment) classification systems.
(3) Endoscopic surveillance of precancerous lesions must be guided by both severity of the histologic finding and the extent and association of other risk factors such as familial gastric neoplasia, autoimmune gastritis, Barrett, and non-eradication of *H. pylori* infection. Endoscopic surveillance is recommended every 3 years in patients with intestinal metaplasia limited to the antrum or gastric body, mainly for patients with familial gastric neoplasia, autoimmune gastritis, or non-eradication of *H. pylori* infection. In patients with chronic atrophic gastritis and/or diffuse intestinal metaplasia, endoscopic follow-up every 1–2 years is recommended.

##### Sources of Evidence

Precancerous conditions such as chronic atrophic gastritis and intestinal metaplasia (including Barrett) represent histologic findings in about one-third of patients on EGDS for dyspeptic symptoms or gastroesophageal reflux [13]. The recommended endoscopic sampling protocol is the updated Sydney system, including five biopsies: two from antrum, two from corpus, and one from the incisura angularis, with additional sampling of suspicious areas.

The degree of atrophy and metaplasia should be assessed by OLGA and OLGIM staging, to identify the risk of neoplasia: patients with stage III and IV are at highest risk of carcinogenesis [14,15]. Major risk factors are age >60, smoking history, and *H. pylori* infection. The American College of Gastroenterology/Canadian Association of Gastroenterology guidelines on dyspepsia management recommend an endoscopic baseline assessment in all patients >60 years with new-onset dyspepsia, especially in those with other risk factors (family history of gastric neoplasia or living in high-risk areas) [16]. A correlation between *H. pylori* infection and chronic atrophic gastritis has been confirmed in two meta-analyses, thus highlighting the significant incidence of chronic atrophic gastritis in serologically positive populations [17,18,19]. In a Korean study, patients achieving *H. pylori* eradication showed a significant regression of previous precancerous conditions [20]. Population screening is recommended in Asian countries, where screening campaigns from age 50, with an endoscopic evaluation or a barium study of the digestive tract every 2 years, have shown significant benefit in early diagnosis and reduced mortality [21,22]. Conversely, in Western countries, population screening is not recommended [23].

Once a precancerous lesion is identified, correct endoscopic follow-up is essential (Table 1), with the timing between endoscopic evaluations based on risk of neoplastic evolution. In patients with chronic atrophic gastritis and intestinal metaplasia, the annual risk for developing gastric neoplasia is 0.1% and 0.25%, respectively; the risk is increased in patients with extensive preneoplastic localization and familial gastric cancers [24,25]. Patients with limited antral intestinal metaplasia therefore require scheduled follow-up in specific conditions such as familial gastric cancers, autoimmune gastritis, or non-eradication of *H. pylori* infection [14]. Endoscopic follow-up is essential in patients with chronic atrophic gastritis and/or intestinal metaplasia extended over the antrum, and timing between endoscopic monitoring should be based on risk factors (familial gastric cancers, autoimmune gastritis, failure to eradicate *H. pylori* infection). In patients with dysplasia without suspicious lesions, an endoscopic evaluation at a tertiary center should be performed initially, with a possible review by expert pathologists to confirm the previous diagnosis and guide the correct endoscopic follow-up [26]. If evident lesions are absent, even at subsequent endoscopic evaluations, a new EGDS must be performed within 6 months in high-grade dysplasia and within 1 year in low-grade dysplasia.

#### 3.2.2. Family History of Gastric Cancer and Hereditary Gastric Cancer (HDGC and Other Syndromes)

**Unanswered question:** How are patients at high risk of familial or hereditary gastric cancer identified? Who should get genetic testing?

##### Statements

(1) Genetic testing is recommended if: there is family history of ≥2 cases of gastric cancer at any age, in first- or second-degree relatives, with ≥1 diffuse histology; a diagnosis of diffuse gastric cancer before age 40 years; or in families with both diffuse gastric cancer and lobular breast cancer with a case identified before age 50 years.
(2) Genetic testing should also be considered for all patients with a diagnosis of gastric neoplasia and a family history compatible with Lynch syndrome, FAP, Peutz–Jeghers, Li–Fraumeni, juvenile polyposis, hereditary breast and ovarian cancer syndrome (germline mutations in BRCA1 or BRCA2).

##### Sources of Evidence

Although most gastric cancers are sporadic, a familial predisposition occurs in approximately 10% of cases. Hereditary gastric cancers account for 1–3% of all cases and include at least three main syndromes: hereditary diffuse gastric cancer (HDGC; with a genetic basis in approximately 40% of HDGC cases); gastric adenocarcinoma and proximal polyposis of the stomach (GAPPS); and familial intestinal gastric carcinoma (FIGC) [27]. Gastric cancer has also been identified in several other hereditary tumor syndromes, including Lynch syndrome, Li–Fraumeni syndrome, familial adenomatous polyposis (FAP), Peutz–Jeghers syndrome, juvenile polyposis, and hereditary breast and ovarian cancer syndrome [27]. Scheduled endoscopic follow-up of these patients is necessary and should be performed at reference centers.

When deciding whether to propose genetic testing, the familial history of gastric cancer, histologic classification, and age of disease onset must be considered. International Gastric Cancer Linkage Consortium criteria to identify patients with suspected HDGC syndrome for genetic testing are: (1) ≥2 cases of gastric cancer at any age in first- or second-degree relatives, with ≥1 diffuse histology; (2) diagnosis of diffuse gastric cancer before age 40 years; (3) families with both diffuse gastric cancer and lobular breast cancer with a case identified before age 50 years. Genetic testing should also be considered for patients with bilateral lobular breast cancer before age 50 years or who have ≥2 relatives with lobular breast cancer before 50 years, a personal or family history of diffuse gastric cancer and cleft palate/lip or in situ signet ring cell (SRC) carcinoma and/or pagetoid spread of SRCs [27]. Only families with HDGC criteria are tested for CDH1 mutations; endoscopic surveillance is offered periodically to people at higher risk in non-HDGC families [28]. All patients with HDGC should ideally receive total gastrectomy, as there is endoscopic follow-up failure in >50% of patients and a >80% risk for developing gastric neoplasia during the course of life [29]. Patients should also be followed up annually with bilateral breast magnetic resonance imaging (MRI) and colonoscopy every 3–5 years [30].

The genetic alterations underlying GAPPS and FIGC are unknown. Clinical criteria to identify those at higher risk of GAPPS are: (1) gastric polyposis restricted to corpus and fundus of the stomach without evidence of duodenal and colorectal polyposis; (2) >100 polyps in the proximal stomach or >30 polyps in a first-degree relative; (3) predominantly fundic gland polyps, some harboring dysplastic regions; (4) an autosomal dominant pattern of inheritance [31].

A diagnosis of FIGC should be considered for a family history of intestinal-type gastric cancer without gastric polyposis. In countries with high incidence rates of gastric cancer the diagnostic criteria for FIGC are close to Amsterdam criteria for Lynch syndrome, whereas in countries with lower incidence rates of gastric cancer, FIGC can be hypothesized if ≥2 first- or second-degree relatives are affected by intestinal-type gastric cancer, one before age 50 years, or if >3 relatives are affected by intestinal-type gastric cancer, regardless of age [32].

### 3.3. Diagnostic and Staging Work-Up

#### 3.3.1. Pathologic Evaluation: Minimum Requirements for Diagnosis and Staging of Gastric Cancer

**Unanswered questions:** What is the minimum biopsy set required for histopathologic diagnosis and staging of gastric cancer? What are the minimum molecular requirements of newly diagnosed gastric cancer? What is the minimum biopsy set for metastatic tissue sampling? What must be reported in an ideal histopathologic report?

##### Statements

1. A histopathologic diagnosis on primary tumor requires at least 6 biopsy samples (optimal 6–8 because of intratumoral heterogeneity).
2. The biopsy specimen report should contain histotype according to WHO classification, expressing the presence and possible percentage of SRC component, Lauren classification, and grading. The surgical anatomopathologic report should contain: macroscopic description of the lesions and sites (primitive/lymph nodes/any other samples), microscopic description including WHO classification, Lauren classification, grading, staging according to the most up-to-date TNM (Tumor-Nodes-Metastasis) version, margin status, lymphovascular and perineural invasion, tumor regression grading (TRG) according to Becker or Mandard after neoadjuvant treatments. Metastatic LNFs (lymph nodes)/total LNFs for every lymph node station should be identified by the surgeon, or at least perigastric lymph nodes (stations 1–7) should be distinguished from extraperigastric lymph nodes (stations 8–12 and 12–16).
3. Microsatellite instability (MSI) should be reported for both operable and metastatic disease, HER2-status assessment is mandatory in metastatic disease and could be reported for localized disease. HER2 status should be determined by immunohistochemistry (IHC) and/or FISH (fluorescence in-situ hybridization) in all patients with metastatic cancer before first-line chemotherapy. HER2 status should preferably be determined on metastatic localization (if a result is not already available) or surgical sample, because of intratumoral heterogeneity, but can also be performed on gastric biopsy, in which case at least five samples must be evaluated for a correct determination.
4. Determining PD-L1 expression is not yet mandatory but could be in the future if immunotherapy approval processes require combined positive score (CPS) for prescriptive purposes.
5. The panel recognizes that NTRK (neurotrophic receptor tyrosine kinase) 1, 2, or 3 genes fusion may be targeted by EMA-approved agnostic drugs such as larotrectinib or entrectinib. These alterations may be identified by immunohistochemistry or nucleic acid-based techniques. However, incidence of gene fusion is low (<1%) in gastroesophageal cancer [33,34,35,36], particularly among Caucasian patients [37], and diagnostic techniques may be not routinely available at all institutions. Therefore, screening of NTRK gene fusion cannot be universally recommended, but is encouraged for pretreated patients without validated therapeutic alternatives [38,39,40,41,42].

##### Sources of Evidence

Considering the intratumoral heterogeneity of gastric cancer, ≥4 biopsies should be performed both on primitive lesion and surrounding area; to achieve a sensitivity of close to 100% 6–8 biopsies are required [34]. The anatomopathologic report should therefore contain the WHO classification with SRC component, Lauren classification, and grading. Correct pathologic staging of surgical specimens is essential, according to the most up-to-date version of the TNM system, as well as reporting the degree of TRG after neoadjuvant treatment. Mandard and Becker systems are useful for prognosis in gastric cancer; however, the prognostic role of TRG as an indicator of response and therefore a surrogate marker of survival is still controversial [36]. A post-hoc analysis of the MAGIC study highlighted that only lymph node status is a significant prognostic factor (overall survival [OS]: hazard ratio [HR] 3.36; 95% CI, 1.70–6.63; *p* < 0.001) [36].

Other globally validated prognostic factors are margin status (R0/R1/R2), histotype, WHO, and Lauren classification subtype (including the percentage of SRCs if present) [35]. The presence of a diffuse histotype (Lauren classification) and of SRC component are recognized prognostic factors, whereas their predictive role is controversial and still being evaluated [35].

In the metastatic setting, evaluating HER2 status is mandatory. Overexpression of HER2 is frequent in proximal cancer (24–32% GEJ; 9.5–18% distal stomach) with an intestinal subtype (16–34% intestinal; 6–7% diffuse) [33,37].

HER2 status is the only validated predictive molecular biomarker in gastric cancer [43,44]. HER2 status must be determined quantitatively by IHC, with equivocal cases confirmed by in-situ hybridization techniques (FISH recommended) [44]. HER2-positivity is defined by the presence of a 3+ or 2+ IHC score with positive FISH, whereas HER2-negative status is characterized by 0, 1+, or 2+ IHC and negative FISH [44]. As part of this determination, the sample and techniques used are fundamental: times and methods of fixation are important so that the analysis is not invalidated (criteria are different for biopsy vs. operation samples), the pathologist must have experience in IHC evaluation, and there should be >6 biopsy specimens because of intratumoral heterogeneity [45,46]. This heterogeneity is amplified in metastatic tissue: a diagnostic biopsy should be performed at the most accessible metastatic site with a new biopsy considered in the event of disease progression for new molecular characterization of the neoplasm [47].

HER2-positive tumors can present primary resistance to target treatments (trastuzumab) or secondary resistance during anti-HER2 therapy owing to the appearance of molecular escape mechanisms, including expression loss of the receptor itself [48,49]. The development during the past decade of multiple molecular classifications for stomach cancer (specifically, The Cancer Genome Atlas [TCGA] and Asian Cancer Research Group) has identified multiple biomolecular prognostic and predictive markers, such as PD-L1 expression, microsatellite instability (MSI), tumor mutational burden, and Epstein–Barr virus (EBV) status [50]: the anatomopathologic report, particularly in metastatic setting, might contain this information.

Incidence of MSI-high cancers varies from 8.2% to 37% in patients with gastric cancer [51]. The prognostic and predictive role of MSI in gastric cancer is still under investigation but in March 2017, the FDA approved the use of pembrolizumab in all patients with metastatic or unresectable tumors with MSI or mismatch repair system protein deficiency after progression on previous chemotherapy and without any additional therapeutic alternatives. Therefore, the determination of microsatellite status should be carried out in all patients progressing after two previous therapeutic lines for metastatic gastric cancer: this determination could also play a role in future perioperative settings. PD-L1 expression seems to be a predictive marker for selecting patients with metastatic gastric cancer and greater benefit from immunotherapy after ≥2 treatment lines (KEYNOTE-059) or in first-line HER2-negative tumors (KEYNOTE-062) [52,53]. CPS should be used to determine PD-L1, as this is the method used in clinical trials [54].

### 3.4. Preoperative Staging

#### 3.4.1. The Role of Endoscopic Ultrasound

**Unanswered question**: What is the role of eco-endoscopy in preoperative staging and response assessment in patients being evaluated for perioperative treatment?

##### Statements

(1) Endoscopic ultrasound (EUS) is the most accurate staging method for defining T parameters; its use is therefore recommended when this impacts the treatment approach (endoscopic resection, upfront surgery, perioperative treatment) (Table 2).
(2) The usefulness of EUS in evaluating response after neoadjuvant chemotherapy or chemoradiotherapy appears limited, so it should not be used for restaging outside clinical trials (Table 2).

##### Sources of Evidence

A Cochrane review confirmed good sensitivity (86%) and specificity (90%) for EUS to discriminate between cT1/2 and cT3-cT4 forms, with greater diagnostic accuracy compared with CT scan and MRI [55]. Similar sensitivity and specificity values are obtained for EUS when discriminating between cT1 and cT2; however, specificity decreases when discriminating between cT1a and cT1b. For lymph node positivity, sensitivity and specificity of EUS are 67% and 83%, respectively.

The role of EUS in assessing response to preoperative treatment is highly controversial: the accuracy of EUS in evaluating T and N parameters is reduced, with poor diagnostic performance, particularly in patients treated with chemoradiotherapy [56,57].

#### 3.4.2. The Role of FDG PET/CT

**Unanswered question**: What is the role of FDG PET/CT in locally advanced disease staging of gastroesophageal cancer (particularly evaluating occult disease: retroperitoneal lymph nodes, peritoneal carcinosis, bone metastases)? Is there any predictive role for FDG PET/CT after neoadjuvant treatment?

##### Statements

(1) Consider PET/CT with [18] F-FDG (fluorodeoxyglucose positron emission tomography/computed tomography) in addition to radiologic imaging in patients with locally advanced gastric cancer when there is no evidence of metastatic disease and in case of suspected secondary lesions at CT scan (Table 2).
(2) FDG PET/CT during treatment or after the end of treatment is useful for identifying patients without any benefit from neoadjuvant treatment; however, this role is marginal outside clinical trials.

##### Sources of Evidence

FDG PET/CT improves diagnostic accuracy compared with CT alone, particularly for identifying occult metastases (retroperitoneal lymph node involvement, bone metastases, and peritoneal carcinosis) to prevent unnecessary surgery [58,59]. The greatest clinical impact of FDG PET/CT is in patients with intestinal or mixed histotype according to Lauren classification, whereas in those with diffuse histotype sensitivity is limited [60,61].

Another possible role of FDG PET/CT is the early evaluation of metabolic response to neoadjuvant treatment (14 days after the first cycle of chemotherapy): metabolic response does not predict pathologic response but identifies a group of patients without any benefit from preoperative treatment who may proceed immediately to resection or receive modified multimodal treatment [62,63,64].

#### 3.4.3. Role of Laparoscopic Surgery

**Unanswered questions:** When should staging laparoscopy and peritoneal cytology be performed? Is there any role for laparoscopic re-evaluation after neoadjuvant treatment?

##### Statements

(1) Staging laparoscopy with peritoneal cytology analysis at diagnosis should be considered in all patients at risk for undiagnosed peritoneal disease (cT3/4 and/or cN+), after collegial discussion, especially in those with suspected peritoneal carcinosis and neoadjuvant therapy candidates, to define the correct therapeutic procedure.
(2) Exploratory/staging laparotomy is not recommended.

##### Sources of Evidence

About 10–20% of locally advanced gastric cancers have occult peritoneal spreading undiagnosed by common imaging tests and the risk is increased among patients with higher clinical T and N stages as well as in specific histologic subsets (such as diffuse-type gastric carcinoma) [65]. Staging laparoscopy is a low-cost procedure with an excellent sensitivity and specificity in peritoneal disease diagnosis [66]. However, the rate of positive cytology in the absence of clinically evident peritoneal disease varies (10–40%) [67,68], and although positive cytology is considered as stage IV disease, the outcome of these patients is different from those with clinically evident peritoneal localizations [69]. In neoadjuvant settings, negative peritoneal cytology after chemotherapy is a prognostic rather than predictive factor, and occult peritoneal progression occurs in some cases [69,70].

#### 3.4.4. Role of Nutritional Support during Early Phases of Disease

**Unanswered question**: What is nutrition screening? What is nutrition counseling? When should enteral nutrition be used?

##### Statements

(1) Validated screening tests for malnutrition should be used in all patients with gastric cancer before any anti-cancer treatment. For pathologic screening tests, the nutritional team must be involved in setting up an intervention.
(2) Nutritional counseling should be the first intervention in pathologic nutritional screening. This aims to maintain adequate weight and quality of life (QoL) during chemotherapy.
(3) Enteral nutrition support in patients treated with radical-intent gastrectomy should be considered in (neo-)adjuvant settings. After surgery, enteral nutrition can be performed through jejunostomy and continued at home.

##### Sources of Evidence

Nutrition screening is the first step in identifying patients who are at risk for nutrition problems or with undetected malnutrition [71]. Several validated screenings MUST (malnutrition universal screening tool), NRS (nutritional risk screening) are available that consider weight loss, body mass index, and feeding limitation [72,73]. For pathologic screening, involvement of the nutritional team is important to perform a more detailed assessment of the state of nutrition and define the degree of malnutrition according to recent European Society of Clinical Nutrition and Metabolism guidelines [73]. The first level of intervention is nutrition counseling by an expert dietician (Table 3). Nutritional advice is a useful tool for optimizing nutrition per os, increasing calories and protein intake, promoting an adequate body weight, and improving patients’ QoL [74,75].

Perioperative nutritional support is essential in candidates for surgery and possible adjuvant CT, particularly for malnourished patients [76,77]. Enteral nutrition (for example, by jejunostomy feeding tube) has been shown to reduce morbidity and duration of hospitalization compared with parenteral support and can be easily continued at home, thus promoting adequate nutritional status 3–6 months post-surgery, with better tolerance of adjuvant chemotherapy [78].

### 3.5. Management of Early Gastric Cancer and Multimodal Approach to Localized Gastric Cancer

#### 3.5.1. Endoscopic Treatment

**Unanswered questions**: What criteria should be used to choose between endoscopic resection and gastrectomy? How should endoscopic follow-up after local dissection be managed?

##### Statements

(1) Endoscopic treatment is only reliable in early gastric cancers with Lauren intestinal histotype, <2 cm, well-differentiated, non-ulcerated, involving only the mucosa (T1a), and without clinical suspected lymph node involvement. Endoscopic resection can only be considered curative when histologic examination of the sample after endoscopic resection confirms all the above criteria with negative margin status (en bloc resection) in absence of vascular-lymphatic infiltration.
(2) Surgical treatment is the therapeutic option for all early gastric cancers that do not meet the criteria described above, or when evaluation of the specimen does not confirm the radicality of the endoscopic resection.
(3) After endoscopic resection of early gastric cancers, endoscopic follow-up after 3 months, 6 months, 1 year, then annually for 5 years is indicated to exclude the presence of locoregional recurrence.

##### Sources of Evidence

**Figure 1:**
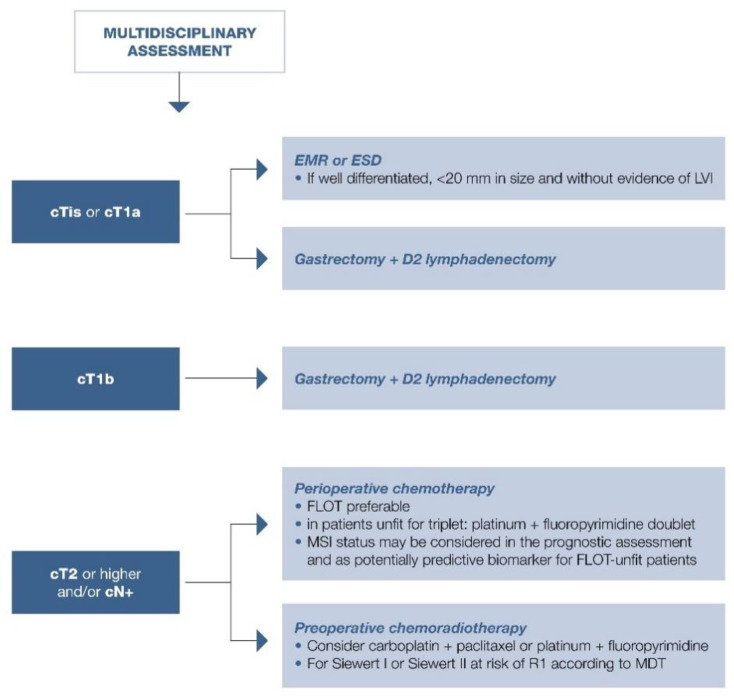
Treatment options for fit patients with resectable gastric or gastroesophageal junction adenocarcinoma. EMR, endoscopic mucosal resection; ESD, endoscopic submucosal dissection; FLOT, 5-fluorouracil/leucovorin, oxaliplatin, and docetaxel; LVI, lymphovascular invasion; MDT, multidisciplinary team; MSI, microsatellite instability.

Endoscopic resection is technically feasible and effective in 81% of early gastric cancers. Long-term disease-free survival (DFS) and disease-specific survival are similar after endoscopy and gastrectomy resection for early gastric cancers (in all groups >95%) [79]. However, risk of relapse is greater after endoscopy (risk ratio, 2.5; 95% CI, 1.3–4.8; *p* = 0.005); metachronous gastric tumor incidence is 7–10% in these patients [80,81]. Endoscopic treatment can be evaluated in patients with early gastric cancers ≤2 cm, without clinically evident lymph node metastases [82,83,84] (Figure 1).

Past series from the East used EUS (in combination with conventional endoscopy and computerized tomography) for the decision-making of gastric cancer patients who were candidates for endoscopic resection or surgery [85]. This approach is undoubtedly of importance, considering mini-invasive surgery as the alternative treatment for early lesions.

Surgical treatment is the preferable option for patients with lesions >2 cm, clinically suspected lymph node metastases, ulcerated and undifferentiated lesions, diffuse histotype, or differentiated lesions but with submucosal invasion [86,87]. Chromoendoscopy allowed the differential diagnosis between early and premalignant lesions with sensitivity, specificity, and AUC greater than simple endoscopy: 0.90 (95% CI, 0.87–0.92), 0.82 (95% CI, 0.79–0.86), and 0.94, respectively [88].

Endoscopic follow-up (together with abdominal CT scan, chest x-ray, and laboratory tests) should be performed at 3 months, 6 months, 1 year, then annually for 5 years after endoscopic early gastric cancer resection [83,89].

#### 3.5.2. Principles of Adequate Surgery

**Unanswered question**: What are the principles of effective surgery?

##### Statements

1. The extent of gastric resection depends on tumor location and the possibility of achieving an adequate free proximal margin.
2. D2 dissection is standard treatment in gastric cancer patients, with the exception of early gastric tumors without nodal involvement.

##### Sources of Evidence

For gastric cancer treatment, surgery is always indicated with the exception of early disease and metastatic tumors. The main role of good-quality oncologic surgery in gastric cancer management is widening the surgical dissection field to achieve a safe circumferential resection margin including a nodal dissection larger than positive lymph node stations [90]. This involves two key principles: primary tumor resection and lymphadenectomy.

Primary Tumor Resection: Total gastrectomy can be avoided if a 5 cm-free resection margin is achieved. However, distal gastrectomy still raises concerns about the radical resection of distal SRC carcinoma due to the risk of underestimated linitis plastic [91]. The extent of gastric resection depends on tumor location and the possibility of achieving an adequate free proximal margin (possibly verified by frozen section examination of the resection line) [91]. In particular with locally advanced disease (generally, after preoperative treatment), splenectomy can be limited to selected tumors located along the greater curvature of the stomach; multivisceral resection is indicated according to the judicious balance between the option of a resection without any residual tumor and risks related to the surgical procedure [92].

Open questions remain about the removal of the peritoneal surface of the omental bursa (bursectomy) for tumors of the posterior gastric wall and total omentectomy. At present, laparoscopic gastrectomy is only validated for distal tumors [93].

Lymphadenectomy: D2 dissection is standard treatment in gastric cancer patients, with the exception of early gastric tumors without nodal involvement; removal of second-level lymph nodes increases the number of retrieved nodes and the accuracy of N staging [94].

As far as it concerns resection margins, 3 issues are of concern for surgeons, including: the minimum amount of macroscopic negative stomach required for a R0 resection, the impact of an extended resection on patient outcomes/quality of life, and other biologic factors including histology [67].

A recent systematic review including some 20,000 patients documented that positive margins after surgery were associated with larger tumor size, T stage, nodal positivity, higher stage, diffuse histology, higher Borrmann type, lymphatic vessel involvement, and total gastrectomy [95].

All of these issues are more than prominent in the era of neoadjuvant treatments and laparoscopic approaches. Still, however, discrepancies in the recommendations provided by NCCN and the Japanese Gastric Cancer Treatment Guidelines based on T stages are currently present.

#### 3.5.3. Sequencing Surgery and Chemotherapy

**Unanswered question**: Perioperative or adjuvant chemotherapy?

##### Statements

(1) In patients with stage II or III gastric carcinoma, perioperative chemotherapy is the standard of care. FLOT (5-fluorouracil [5-FU], oxaliplatin, and docetaxel) is first choice in fit patients.
(2) Patient compliance to preoperative treatment is higher than postoperative treatment.
(3) Preoperative chemotherapy does not increase morbidity and early post-surgery mortality: FLOT is not associated with greater risk for post-surgical complications or mortality than ECF (epirubicin, cisplatin, and 5-FU) or ECX (epirubicin, cisplatin, and capecitabine).
(4) After surgery without any preoperative treatment, an adjuvant therapy with fluoropyrimidine and/or oxaliplatin is recommended.
(5) In the adjuvant setting after upfront surgery for pathologic stage II–III disease, the combination of fluoropyrimidine and oxaliplatin should be considered the preferred option in all patients with adequate recovery after resection.
(6) All patients undergoing surgery for stomach cancer should receive postoperative treatment, regardless of prognostic factors and pathologic response.

##### Sources of Evidence

Key evidence for the use of perioperative chemotherapy is provided by the MAGIC and ACCORD 07/FFCD trials [96,97]. Data from these and 12 other studies that compared perioperative treatment versus surgery alone were analyzed in a 2013 Cochrane meta-analysis including 2422 patients with gastroesophageal neoplasm [98]. Perioperative chemotherapy, regardless of the addition of radiotherapy, had a significant OS benefit (HR = 0.81; 95% CI, 0.73–0.89) with an absolute death risk reduction of 9% [98].

Subsequently, three important prospective trials were published demonstrating:

(1) There is no difference in OS, but less toxicity, with two preoperative cycles of fluoropyrimidine and cisplatin compared with four preoperative cycles with ECX in patients with esophageal and Siewert I-II GEJ cancers [99].
(2) There is no benefit from adding bevacizumab to perioperative ECX chemotherapy: 3-year overall survival 50.3% with chemotherapy versus 48.1% with addition of bevacizumab [100].
(3) Pathologic regression grade/response (<pT1: 15% vs. 25%) and overall survival (50 months vs. 35 months; HR = 0.77; 95% CI, 0.63-0.94; *p* = 0.012) benefit from perioperative FLOT compared with the ECF/ECX combinations. OS at 2–3 and 5 years was 59%, 48%, and 36% with ECF/ECX compared with 68%, 57%, and 45% with FLOT [101]. Similar rates of post-surgical complications with FLOT and ECF/ECX were reported (51% vs. 50%). Median duration of hospitalization was also similar in the two arms (15 vs. 16 days), as were reoperation rates (10% vs. 11%), and deaths within 30 days (2% vs. 3%) and within 90 days (5% vs. 8%).

The use of adjuvant chemotherapy in gastric carcinoma is supported by various meta-analyses including one by the GASTRIC group, analyzing 3,838 patients enrolled in 17 randomized clinical trials, in which there was a relative reduction in mortality at 5 years of 18% (HR = 0.82; 95% CI, 0.76–0.90) compared with surgery alone with OS increased from 49.5% to 53% [102]. Polychemotherapy regimens are commonly used in Asian populations, based on CLASSIC trial results comparing adjuvant Xelox (capecitabine + oxaliplatin) with surgery alone: 5-year disease-free survival (DFS) increased from 53% to 68% (HR = 0.58; 95% CI, 0 47–072; *p* < 0.0001) and OS improved from 69% to 78% (HR = 0.66; 95% CI, 0 51-0 85; *p* = 0.0015) [103]. Similar evidence is lacking for European and US populations. In JACCRO GC-07, addition of docetaxel to S1 alone (S1 is a fluoropyrimidine, similar to 5-FU and capecitabine) in the adjuvant setting demonstrated significantly improved 3-year DFS post-surgery: S1 + docetaxel 66% versus S1 50% (HR = 0.63; 95% CI, 0.400–0.998; *p* = 0.001) [104]. A network meta-analysis analyzed 5620 patients enrolled in 11 randomized trials, demonstrating a significant survival benefit versus surgery alone from adjuvant treatment with 5-FU + radiotherapy, S1, Xelox: 5-FU + radiotherapy, S1 and Xelox (HR = 0.75; 95% CI, 0.63–0.89; 0.63; 95% CI, 0.52–0.76; and 0.66; 95% CI, 0.51–0.85, respectively), with no clear difference between adjuvants [105].

Some evidence in Asian populations (Resolve trial and Prodigy trial) supports the role of perioperative treatment, although no Phase 3 trials have been published comparing perioperative and adjuvant chemotherapy in stage II–III gastric cancer [106].

The completion rate of preoperative therapy in the above-mentioned Phase 3 studies was approximately 90% [99,100,101]. This percentage drops dramatically when considering preoperative and postoperative treatment (35–45%) [99,100,101]. In most recent adjuvant trials with combination regimens, the percentage of patients completing treatment is generally <70% [99,100,101]. In the SAKK 43/99 study comparing preoperative TCF (docetaxel, cisplatin, and fluorouracil) with postoperative TCF, the percentage of patients completing the four cycles was double in the preoperative therapy arm versus postoperative (75.8% vs. 37.5%) [107].

In MAGIC, the two treatment arms (perioperative chemotherapy and surgery) were associated with similar postoperative complication rates (45.7% vs. 45.3%), with equal mortality within 30 days of surgery (5.6% vs. 5.9%) and the same duration of recovery (median: 13 days): only 10 patients did not start chemotherapy owing to postoperative complications [96]. Similar results have been reported in the French FNCLCC-FFCD [97]. Postoperative morbidity was 25.7% with chemotherapy compared with 19.1% with surgery, with mortality rates of 4.6% and 4.5%, respectively.

A meta-analysis published in 2016 analyzed 11 studies in China and Japan, including 1240 patients with gastric cancer, demonstrating a survival advantage of perioperative polychemotherapy compared with surgery followed by adjuvant chemotherapy (HR = 0.74; 95% CI, 0.61–0.89, *p* < 0.05) [108]. In a network meta-analysis including 4187 patients enrolled in 14 randomized studies, perioperative taxane-based polychemotherapy improved survival compared with surgery alone (HR = 0.58; 95% CI, 0.38–0.91; *p* < 0.05) and compared with surgery followed by adjuvant treatment (HR = 0.62; 95% CI, 0.42–0.93; *p* < 0.05) [109]. In terms of adjuvant chemotherapy regimens, Xelox provided the best survival benefit vs. other chemotherapy regimens: OS (HR = 0.47; 95% CI, 0.28–0.80) and relapse-free survival (HR = 0.40; 95% CI, 0.24–0.64) [109].

In a subsequent network meta-analysis including 7235 patients, perioperative taxane-based treatment with FLOT proved the most effective option for survival, followed by adjuvant chemotherapy with platinum and 5-FU, and monotherapy with S1. In subsequent subgroup analyses, chemotherapy with capecitabine and platinum proved to be the best regimen in Asian populations, whereas chemotherapy with FLOT was better in European populations [110].

**Figure 2:**
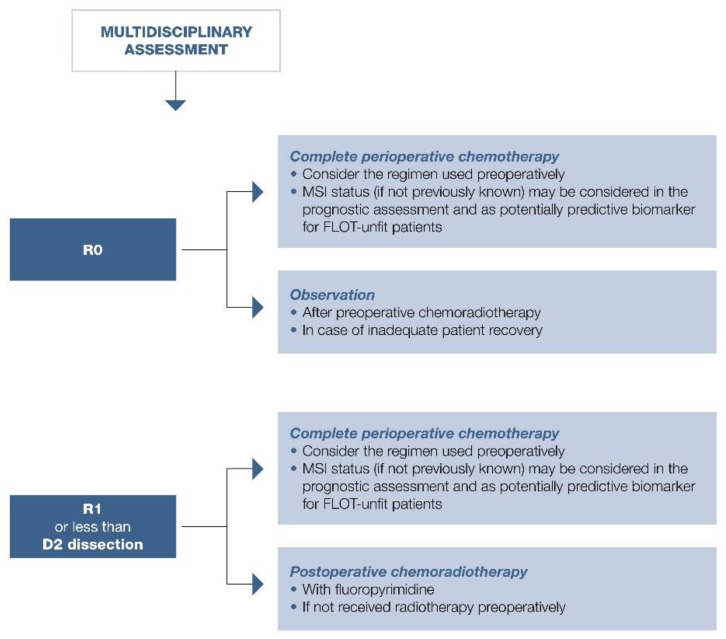
Treatment options for fit patients with resectable gastric or gastroesophageal junction adenocarcinoma already treated with preoperative therapy and surgery. FLOT, 5-fluorouracil/leucovorin, oxaliplatin, and docetaxel; MSI, microsatellite instability.

**Figure 3:**
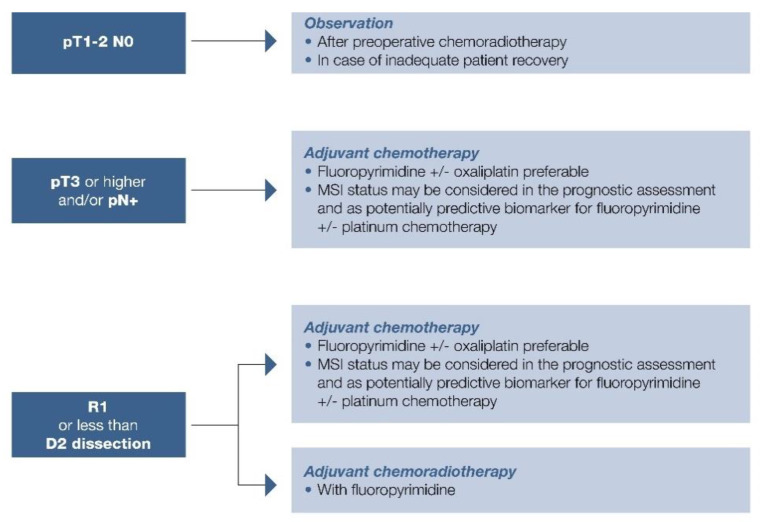
Treatment options for fit patients with resected gastric or gastroesophageal junction adenocarcinoma. MSI, microsatellite instability.

Suggested approach in summarized in Figure 1, Figure 2 and Figure 3.

**Unanswered question**: Is it time for a histotype-driven treatment?

##### Statement

The presence of SRCs at diagnosis does not change the indication of perioperative treatment in patients with stage II or III gastric cancer.

##### Sources of Evidence

Lauren’s classification has identified two distinct subtypes based on histopathologic, etiologic, biological, and prognostic characteristics: intestinal and diffuse (with or without SRCs) [111]. Conversely, WHO classification defines five main histotypes: papillary, tubular, mucinous, poorly cohesive, and mixed [112]. There is no evidence of a predictive role for these different histotypes. A recent meta-analysis, including data from 73 published studies of 61,000 patients, confirmed that patients with diffuse-type histotype have worse prognosis compared with patients with intestinal subtypes (HR = 1.23; 95% CI, 1.17–1.29; *p* < 0.0001) in loco-regional and advanced-stage disease (HR = 1.21; 95% CI, 1.12–1.30, *p* < 0.0001 and HR = 1.25; 95% CI, 1.046–1.50; *p* = 0.014), regardless of any treatment added to surgery [35].

In a French retrospective analysis of 924 patients with SRC neoplasia from 1997 to 2010, 171 patients received preoperative chemotherapy with cisplatin and 5-FU or cisplatin-5-FU-epirubicin and 753 had upfront surgery [113]. Preoperative chemotherapy was detrimental: median OS was 12.8 months versus 14.0 months in patients receiving only surgery (*p* = 0.043) [113].

A subsequent German analysis confirmed the correlation between SRC component and inadequate surgery (R1) associated with worse histopathologic response (<10% of residual neoplasia in 16.3% vs. 28.9% of patients with intestinal histotype; *p* < 0.001) and increased risk for peritoneal dissemination (*p* < 0.001) in patients with resectable gastric cancer treated with preoperative chemotherapy (CF-based, with taxanes or epirubicin) [114].

In FLOT-4, the regression grade benefit observed with docetaxel (TRG = 1) was limited to intestinal histotype (23% with FLOT and 10% with ECF/X) and not confirmed in diffuse histotype (3% in both arms) [101]. Subsequent analysis of the SRC carcinoma group showed that FLOT was effective in these patients (HR for survival: 0.74 vs. 0.79 in the intestinal subgroup, log-rank *p* = 0.005). These data were confirmed by a French trial with TEFOX (docetaxel, oxaliplatin, and 5-FU) in patients with locally advanced/unresectable and metastatic gastric neoplasia [115]. In 65 patients with SRC carcinoma, objective response rate (ORR) was 66.1% and disease control rate (DCR) was 87.6% [107]. In this context, data from the Phase 2 PRODIGE 19 study were reported at ASCO 2019: 83 patients with SRC were randomized to upfront surgery followed by adjuvant chemotherapy or perioperative ECF chemotherapy [116]. Resection rates and median survival were higher with perioperative chemotherapy: R0, 88%/78%; 2-year OS, 60%/53.5%; median survival, 39/28 months (HR = 0.71; 95% CI, 0.40–2.64) [116].

All these data highlight the need for an intensive perioperative chemotherapy treatment (if feasible, taxane-based) for patients with SRC carcinoma.

**Unanswered question**: Is there a role for molecular classification?

##### Statement

MSI status could be used for the selection of an appropriate therapeutic strategy: specifically, perioperative chemotherapy versus upfront surgery.

##### Sources of Evidence

The TCGA research network analyzed the genomic profile of 295 gastric malignancies, identifying four molecular subtypes of gastric cancer: neoplasms with MSI; neoplasms related to EBV infection; neoplasia with chromosomal instability (CIN); and genomically stable neoplasms (GS) [109]. The first group (22% of those analyzed) is characterized by hypermethylation of CIMP or MLH1 and, together with the related EBV subtype (9% of analyzed neoplasms) by the greatest benefit from immunotherapy. The CIN subtype (50% of cases) is mainly characterized by tumors with an intestinal variant according to Lauren classification and with gene alterations such as mutations involving in particular TP53 [50]. Finally, the GS subtype seems to overlap the diffuse histotype according to Lauren classification with alterations of CDH-1 and RHOA [50].

Currently, the molecular classification proposed by the TCGA research network, and the subsequent classification proposed by Cristescu et al. [117] are only used in clinical trials and not in clinical practice. The EBV subtype has the best prognosis, whereas patients with the CIN subtype gain the greatest benefit from adjuvant chemotherapy (HR = 0.39; 95% CI, 0.16–0.94; *p* = 0.03), despite those with the GS subtype being characterized by poor benefit from chemotherapy and the worst prognosis (HR vs. no chemotherapy 0.83; 95% CI, 0.36–1.89; *p* = 0.65) [117,118,119].

MSI expression was more frequent in elderly patients with distal gastric carcinoma and intestinal histotype according to Lauren, with a strong prognostic impact on stage I-III gastric cancers [120,121]. These data were confirmed by a meta-analysis of 18,612 patients enrolled in 48 trials with an OS benefit reported with MSI (HR = 0.69; 95% CI, 0.56–0.86; *p* < 0.0001) [122]. Further, a subgroup analysis by Pietrantonio et al. using data from MAGIC, CLASSIC, ITACA-S, and ARTIST trials highlighted the detrimental effect of chemotherapy in patients with MSI: those with MSI-low/MSS gastric cancer benefitted from chemotherapy with 5-year DFS of 57% compared with 41% in patients treated with surgery alone (HR = 0.65; 95% CI, 0.53–0.79); 5-year OS was 62% versus 53% (HR = 0.75; 95% CI, 0.60–0.94) [123]. Conversely, those patients with MSI-high gastric cancer did not benefit from chemotherapy: 5-year DFS, 70% versus 77% in the MSS group (HR = 1.27; 95% CI, 0.53–3.04), and 5-year OS, 75% versus 83% (HR = 1.50; 95% CI, 0.55–4.12) [123]. The effect of taxane-based chemotherapy (FLOT) is awaited. Role of MSI in localized disease is summarized in Figure 1, Figure 2 and Figure 3. 

MSI status is also an important predictive tool in advanced disease: in a subgroup analysis of KEYNOTE 059, 9 patients with MSI treated with pembrolizumab had an ORR of 57.1% and DCR >70%, compared with an ORR of 9% and DCR of 22.2% in non-MSI-high patients [53]. These results were confirmed in KEYNOTE 061 (ORR = 46.7%) and KEYNOTE 062 (ORR = 57%) and in a recent Phase 2 Korean study (ORR = 85.7% in MSI-high and ORR = 100% in EBV patients) [54,124,125].

**Unanswered question**: What is the treatment of choice for stage IB gastric cancer (T2N0M0)?

##### Statement

(1) Perioperative chemotherapy can be considered first choice for patients with stage IB gastric cancer after multidisciplinary discussion about the site of the primary tumor, the characteristics of the patient (age, performance status [PS], comorbidities), and the biology of the tumor (histotype, MSI).

##### Sources of Evidence

The choice of the appropriate treatment for patients presenting with stage IB tumor (T2N0M0) is still controversial and requires multidisciplinary evaluation. Although Italian Society of Medical Oncology guidelines recommend the use of perioperative treatment for patients who have T3 and/or N+ tumors, ESMO and NCCN guidelines recommend the use of a perioperative strategy as treatment of choice in patients with muscular invasion gastric cancer (>T1) regardless of clinical lymph node involvement. These recommendations are based on the results of the main Phase 3 trials in perioperative treatment (MAGIC, ACCORD, and FLOT-4) [96,97,101]. Although MAGIC only enrolled patients from stage II onwards and ACCORD did not report any subgroup analyses on stage at diagnosis, FLOT-4 clearly demonstrated a survival benefit with perioperative strategy in all subgroups, including patients with cT1/2 (16%) and cN- (21%) tumors [96,97,101].

In GEJ tumors, the CROSS trial demonstrated survival benefit of chemoradiotherapy with carboplatin and paclitaxel in addition to surgery in all patient subgroups, including cN0 patients (32% of the study population) [126]. However, this benefit derived mostly from patients with squamous histology.

**Unanswered question**: When should adjuvant chemotherapy be started?

##### Statement

Completion of adjuvant treatment is critical to reducing risk for recurrence. Adjuvant chemotherapy should be undertaken within 8 weeks of surgery. If treatment is delayed toallow recovery of the patient for better compliance, this should still be considered within 12 weeks of surgery.

##### Sources of Evidence

The interval between surgery and adjuvant chemotherapy (time to adjuvant) in patients with gastric cancer can be influenced by the long postoperative course characterized by post-surgical complications and by the need for proper re-feeding of patients, and a number of studies have emphasized the importance of completing adjuvant treatment (as part of a perioperative program or absence of preoperative chemotherapy) [127,128,129,130].

In the Italian ITACA-S trial, chemotherapy was stopped in 201 patients (18.8%), whereas it was completed without decreasing drug dosages only in a quarter of patients: discontinuation of treatment impacted DFS (HR = 0.74; 95% CI, 0.60–0.91; *p* = 0.004) and OS (HR = 0.69; 95% CI, 0.55–0.86; *p* = 0.001) on multivariate analysis [127]. In the same study, patients with an extended interval between surgery and adjuvant chemotherapy reported benefits for DFS (HR = 0.95; 95% CI, 0.89–1.00; *p* = 0.05) and OS (HR = 0.91; 95% CI, 0.86–0.97; *p* = 0.004). These data were confirmed by a retrospective analysis of 7942 patients in which three groups of patients undergoing adjuvant chemotherapy within 8 weeks of surgery, 8–12 weeks after surgery, and >12 weeks after surgery had the same benefit from adjuvant treatment [128]. In contrast, two subsequent Korean analyses showed that prolonging the chemotherapy-free interval beyond 2 months in one study and over 28 days in the second seems to have a detrimental effect on the risk of recurrence [129,130].

These studies were included in a meta-analysis by Petrelli et al.: initiation of adjuvant treatment beyond 8 weeks after surgery was detrimental on survival (HR = 1.2; 95% CI, 1.04–1.38; *p* = 0.01) [131].

**Unanswered question**: When should adjuvant chemoradiotherapy be considered?

##### Statements

(1) In patients with stage II/III gastric cancer undergoing suboptimal surgical (<D2) or with R1, adjuvant chemoradiotherapy should be considered as the first therapeutic option.
(2) In patients with stage II/III gastric cancer undergoing radical surgery, adjuvant chemoradiotherapy can be considered after adjuvant chemotherapy in pN+ patients, after multidisciplinary discussion.

##### Sources of Evidence

A randomized study published in 2001 (INT 0116) on 556 patients demonstrated a survival benefit for patients treated with concomitant postoperative 5-FU chemoradiotherapy compared with surgery alone [132]. The combined approach was superior both for 3-year DFS (48% vs. 31%; *p* < 0.001) and OS (50% vs. 41%; HR = 1.35; 95% CI, 1.09–1.66; *p* = 0.05) [133]. These results were confirmed in the 10-year update. However, INT 0116 was criticized for the low quality of the surgery, evidenced by the low probability of survival and high percentage of local recurrences attributed to the very small number of D2 resections compared with the prevalence of D0 resections (D2, 10%; D1, 36%; D0, 54%). A retrospective study published in 2005 of 544 Asian patients undergoing D2 resection followed by chemoradiotherapy had a 20% mortality reduction (HR = 0.80; *p* = 0.02) compared with a group of 446 undergoing D2 resection in the same period [134].

The role of adjuvant chemoradiotherapy in radical resected patients was evaluated in ARTIST: patients who underwent D2 resection received chemotherapy with CDDP and capecitabine for six cycles or two cycles of the same chemotherapy followed by concomitant chemoradiotherapy [135]. With a median follow-up of 53.2 months, there was no significant advantage of DFS with chemoradiotherapy compared with chemotherapy (78.2% vs. 74.2%; *p* < 0.086). These results were confirmed by an analysis in the IB subgroup (according to AJCC 2002) [136]. However, DFS was superior to chemoradiotherapy in the pN+ subgroup (77.5% vs. 72.3%; *p* = 0.036). Further, in the latest update of the study, there was a significant benefit for locoregional recurrences in the chemoradiotherapy arm (7% vs. 13%; *p* = 0.03), that was more marked for the pN+ subgroup (*p* = 0.009) [137].

This result led the group to undertake ARTIST 2, aimed at testing the role of chemoradiotherapy only in pN+ patients undergoing D2 resection. In an interim analysis there was no reduction in risk of recurrence from the addition of radiotherapy to polychemotherapy with S1 and oxaliplatin (HR = 0.910; *p* = 0.667) [138].

#### 3.5.4. Treatment of Siewert II Adenocarcinoma

**Unanswered question**: Should perioperative chemotherapy or preoperative chemoradiotherapy be the preferred option in patients with Siewert II adenocarcinoma?

##### Statement

(1) In patients with Siewert II adenocarcinoma, while acknowledging the fundamental role of a multidisciplinary discussion for every single situation, perioperative chemotherapy with FLOT is preferable, reserving preoperative chemoradiotherapy for patients at high risk for R1 resection and local recurrence.

##### Sources of Evidence

Two studies have compared chemoradiotherapy and preoperative chemotherapy in patients with GEJ adenocarcinoma, although both closed early owing to poor enrollment [139,140]. In the first, patients were randomized to receive cisplatin and 5-FU chemoradiotherapy (39 patients) or chemotherapy alone (36 patients) [137]. Median OS was 32 versus 29 months (*p* = 0.83) but significant regression was documented in the combination arm (pathologic complete response [pCR]: 13% vs. 0%; *p* = 0.02) [140]. POET randomized 126 patients to chemoradiotherapy or chemotherapy alone, with a survival benefit at 3 and 5 years in the first group (46.7% and 39.5% vs. 26.1% and 24.4%, respectively) at the expense of increased postoperative mortality (10.2% vs. 3.8%; *p* = 0.26) [139]. Thus, there was a trend for OS in favor of preoperative chemoradiotherapy (HR = 0.65; 95% CI, 0.42–1.01; *p* = 0.055). The advantage of the combination was also confirmed for pCR (15.6% vs. 2.0%; *p* = 0.03), R0 resection rates (15.4% vs. 4.1%), and pCR at lymph node level (pN0, 64% vs. 38.8%; *p* < 0.001).

In a meta-analysis of 22 studies and 18,260 patients, the benefit of preoperative chemoradiotherapy compared with chemotherapy alone was evident only in pCR rate (odds ratio [OR] = 2.8 in favor of combined treatment; 95% CI, 2.27–3.47; *p* < 0.001) and reduction of local recurrence (OR = 0.6; 95% CI, 0.39–0.91; *p* = 0.01) with no benefit in distant relapses or survival [141].

In CROSS, survival benefit at 5 years from addition of preoperative chemoradiotherapy to surgery in the entire cohort (squamous cell carcinoma and adenocarcinoma) was 14% [126]. Of the 366 patients enrolled, 88 had gastroesophageal Siewert I-II adenocarcinoma: in this group of patients, median OS increased from 27.1 months to 43.2 months [126].

In FLOT-4, approximately 56% of patients enrolled had a GEJ malignancy (Siewert I–III); the OS benefit in the study (5-year survival increased by 9% with FLOT vs. ECF/ECX) was also observed in the subgroup of patients with pCR, with results comparable to preoperative chemoradiotherapy treatment (16% in FLOT-4 vs. 14% in POET) [101].

Neo-AEGIS and ESOPEC are currently enrolling patients with adenocarcinoma of the esophageal/GEJ to perioperative treatment with ECF (Neo-AGIS) or FLOT (ESOPEC) or neoadjuvant chemoradiotherapy according to CROSS scheme.

### 3.6. Treatment of Elderly Patients with Locally Advanced Resectable Disease

#### 3.6.1. (Neo-)Adjuvant Therapy

**Unanswered questions**: Does age influence treatment choice in locally advanced resectable disease? Is there a role for multidimensional geriatric assessment (MGA)?

##### Statements

1. MGA allows the identification of frail patients who are at higher risk for complications after gastrectomy.
2. The role of MGA in determining medical treatment is less established in resectable disease compared with the palliative setting. However, MGA might also help the MDT in personalizing treatment approaches when the aim of treatment is curative.
3. Age does not impact perioperative chemotherapy benefit over surgery alone or the benefit of FLOT over ECF/ECX.
4. High-grade toxicities with FLOT are more common in the elderly. Accurate patient selection is therefore needed with taxane-based triplet regimens.

##### Sources of Evidence

Both MAGIC and FLOT-4 defined elderly patients as those 70 years or older [93,121]. A significant proportion of elderly patients have been enrolled in each study (20.4% and 24%, respectively). There is no significant interaction between treatment effect for the experimental arm and age in these studies, with HRs favoring experimental treatment in this subset. Indeed, in locally advanced and metastatic disease, the FLOT65+ study reported a higher incidence of grade 3-4 toxicities with FLOT compared with FLO [137].

MGA is an important instrument in the palliative setting, useful for identifying frail patients at higher risk of toxicity and rapid deterioration [139,140]. Studies investigating MGA in locally advanced resectable disease as a tool to inform medical (neo-)adjuvant treatment are lacking. Evidence of frailty at MGA is associated with increased risk for postoperative complications and readmission within 1 year of resection [142,143].

#### 3.6.2. Treatment of Locally Advanced Unresectable and Metastatic Disease

##### First-Line Therapy

**Unanswered questions:** Is there a preferred first-line regimen in advanced gastric cancer? Which clinical parameters (e.g., PS, comorbidities, previous treatments, age) should be considered in the definition of optimal first-line therapy? What are the main biological parameters (e.g., HER2 and MSI status, EBV+, PDL-1 expression) when defining optimal first-line therapy? How should the aim of treatment guide treatment choice in first line? Is it possible to define oligometastatic disease? Which of the following elements should be considered in the treatment of advanced disease: rebiopsy on sites of recurrence, re-assessment of HER2 status on sites of recurrence, or liquid biopsy to guide treatment choice and monitor treatment efficacy?

##### Statements

(1) Patient PS, comorbidities, and disease burden are all factors that should be considered in the choice of first-line therapy.
(2) Among molecular parameters investigated, only HER2 status has been validated as a predictive biomarker for choice of first-line therapy.
(3) Platinum plus fluoropyrimidine doublet chemotherapy regimens represent standard of care in this setting.
(4) Triplet chemotherapy, mainly FLOT, is preferable for selected fit patients (i.e., with Eastern Cooperative Oncology Group [ECOG] PS 0–1, adequate organ function) with either locally advanced unresectable disease or high tumor burden.
(5) While deciding optimal first-line chemotherapy in individual patients, a cautious assessment of residual toxicity from (neo-)adjuvant therapies as well as time interval between treatment interruption and evidence of recurrence is recommended.
(6) For fit patients experiencing progression soon after (i.e., ≤6 months of completion) or during adjuvant treatment with platinum plus fluoropyrimidine, the combination of paclitaxel plus ramucirumab is the preferred choice.
(7) For fit patients experiencing progression soon after (i.e., ≤6 months of completion) or during adjuvant treatment with docetaxel, FOLFIRI or ramucirumab should be considered. If recurrence occurs >6 months after completion of adjuvant therapy, paclitaxel plus ramucirumab should be considered.

##### Sources of Evidence

Several meta-analyses have evaluated the optimal first-line chemotherapy regimen in advanced gastric cancer. Wagner et al. analyzed 64 trials comparing different first-line chemotherapy regimens with an active comparator or best supportive care (BSC) alone [144]. Chemotherapy significantly prolonged OS compared with BSC, and combination chemotherapy was superior to single-agent chemotherapy, although the advantage of polychemotherapy was modest. A subsequent network meta-analysis tried to determine the preferred first-line regimen among different options available [145]: fluoropyrimidine-based doublets with oxaliplatin, irinotecan, or docetaxel were preferred on the basis of survival and safety profiles, whereas FLOT was the most effective and well tolerated triplet compared with conventional doublets. These findings were confirmed in a subsequent conventional meta-analysis of three versus two agents, which confirmed that three-drug regimens containing fluoropyrimidines (vs. no fluoropyrimidines), cisplatin (vs. no cisplatin), and docetaxel (vs. no docetaxel) were associated with a significant OS advantage over doublets [146].

Chau et al. first investigated the main clinical prognostic determinants in 1080 patients with advanced esophagogastric cancer from three randomized, controlled trials [147]. Four poor prognosis parameters were identified: PS > 2, liver metastases, peritoneal carcinomatosis, and baseline alkaline phosphatase >100 U/L. Scores based on these variables stratified patients into three risk categories: low (no risk factors), moderate (1–2 risk factors), and high (3–4 risk factors), associated with significantly different OS. The same group subsequently validated the prognostic score on the separate REAL-2 database [148].

Similar analyses have also been conducted using data from patients not included in randomized, controlled trials [149,150]. PS, peritoneal metastases, and alkaline phosphatase were confirmed as poor prognostic determinants [149,150]. Wang et al. developed a prognostic score comprising presence of on-site primary tumor, number of disease sites, bone or liver metastases, and neutrophil-to-lymphocyte ratio [150]. Again, the score identified three risk groups with significantly different OS.

None of these studies investigated the role of age. As more effective and intensive doublet and triplet regimens are used, an increased interest has been focused on elderly patients, to establish tolerability and efficacy of modern combinations in this underrepresented population in clinical trials. Age has been found as a main determinant of treatment choices in routine clinical practice [151]. In FLOT65+ [152], FLOT was associated with higher relative risk (RR) than 5-FU plus oxaliplatin, but grade 3/4 toxicity also increased in patients aged ≥65 years. In particular, triplet was associated with higher rates of neutropenia, leucopenia, diarrhea, and nausea. A subset analysis did not confirm the benefit of triplet among patients >70 years.

No definitive data are available on first-line treatment choice after a (neo-)adjuvant regimen. This is partly due to the high proportion of patients with synchronous distant metastases or unresectable disease at diagnosis and the rapid deterioration in many patients after recurrence [153]. Even if confirmation from prospective data is lacking, disease progression after ≥6 months of completion of (neo-)adjuvant systemic therapy (generally comprising a platinum derivative and fluoropyrimidine) is generally regarded as an adequate period to also consider the same agents in first line [154,155,156]. The main challenge is represented by those patients progressing within 6 months of completion of adjuvant therapies: in such cases, disease can be considered refractory to the agents used in the curative setting, and second-line therapy is considered adequate. In RAINBOW, 76% of patients enrolled experienced disease progression within 6 months of fluoropyrimidine plus platinum (69% received this treatment as first-line) [157]. The benefit of paclitaxel plus ramucirumab was also confirmed in the subset of patients with progression-free survival (PFS) <6 months in the previous line, which also included patients progressing during adjuvant treatment. Therefore, in early progression after platinum plus fluoropyrimidine, paclitaxel plus ramucirumab may be considered a preferred option. If progression occurs during or early after perioperative FLOT, even with the lack of adequate literature, irinotecan (either alone or with 5-FU) [158] or ramucirumab monotherapy (particularly for patients with initial PS deterioration) can be considered [159]. First-line 5-fluorouracil plus irinotecan (FOLFIRI) is among the accepted doublets in randomized trials and meta-analyses [160]: therefore, this regimen should be considered, particularly in patients with residual toxicity from adjuvant therapies (e.g., peripheral neurotoxicity), which may impact QoL.

Trastuzumab is the only approved targeted first-line agent. Its label was updated based on the results of the Phase 3 ToGA trial [46], which demonstrated that adding trastuzumab to first-line cisplatin plus fluoropyrimidine resulted in increased OS, PFS, time to progression (TTP), and RR compared with placebo. Based on these data, trastuzumab is now standard-of-care in combination with platinum plus fluoropyrimidine doublet for patients with HER2-positive disease, defined as HER2 3+ or HER2 2+ at IHC with gene amplification by FISH.

**Figure 4:**
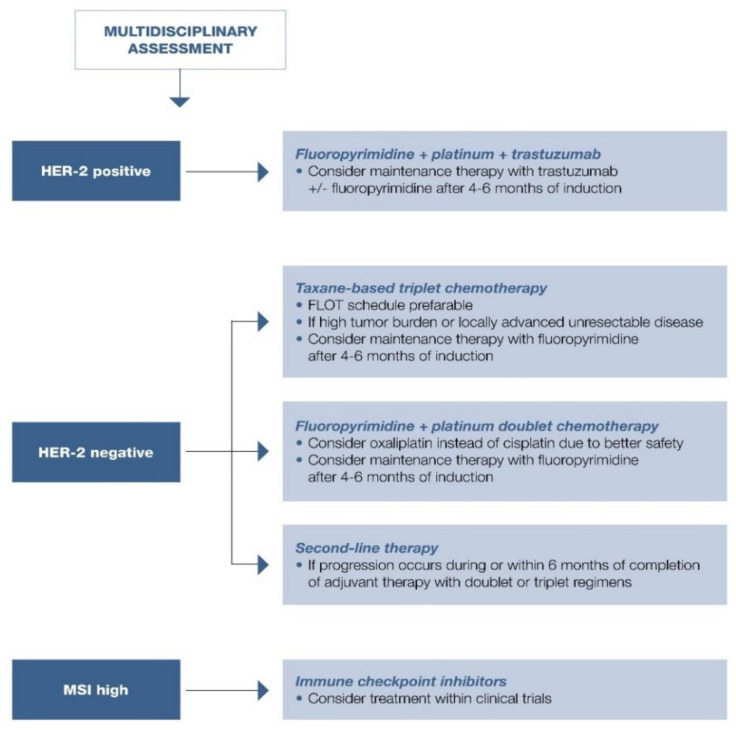
First-line treatment options for fit patients with metastatic or unresectable gastric or gastroesophageal junction adenocarcinoma. FLOT, 5-fluorouracil/leucovorin, oxaliplatin, and docetaxel; MSI, microsatellite instability.

First-line treatment options are summarized in Figure 4.

Other than HER2 status, no other molecular biomarker has entered clinical practice. KEYNOTE-062 demonstrated that pembrolizumab monotherapy is non-inferior to first-line chemotherapy for OS in patients with PDL-1-positive disease (defined as CPS ≥1), whereas adding pembrolizumab to chemotherapy did not result in increased OS [54]. In a subgroup analysis of patients with PDL-1 CPS ≥10, single-agent pembrolizumab was superior to chemotherapy for OS. KEYNOTE-062 results are consistent with KEYNOTE-061 and KEYNOTE-059 [53,119], and support further research into PDL-1 as potential biomarker for pembrolizumab efficacy. KEYNOTE-062 exploratory analyses into the role of MSI status clearly show that patients with MSI-high disease derive the greatest benefit from pembrolizumab, both as single agent and in combination with chemotherapy. Future studies will clarify the role of MSI and EBV status in determining benefits of immunotherapy, as these parameters seem the most promising predictive biomarkers for anti-PD(L)1 agents [118].

The need for HER2 reassessment at recurrence is a matter of research, but is not mandatory in routine practice [156].

HER2 expression is heterogeneous in gastric cancer [161] and this feature might also impact treatment efficacy with anti-HER2 agents [162]. However, availability of surgical tissue from previous gastrectomy should increase the accuracy in HER2 evaluation compared with limited biopsy samples [163,164]. Therefore, HER2 assessment on surgical samples (if already available) is regarded as adequate to select candidates for trastuzumab. For patients with synchronous distant metastases, biopsy of both primary tumor and metastatic sites are acceptable for HER2 evaluation, provided all quality requirements for pathologic and molecular assessment are met. Some series have highlighted the possibility of discordant results between primary tumor and metastasis HER2 expression. The GASTHER-1 prospective study found that rescued HER2-positivity rate of 8.7% could be recognized with additional sampling among patients whose tumor was initially defined as HER2-negative, particularly among tumors with non-diffuse histology and HER2 2+ [165]. Moreover, a HER2-positivity rate of 5.7% was recognized by sampling of metastases in initially HER2-negative patients; this was associated with metastatic site (liver lesions being more frequently HER2-positive). Notably, patients with rescued HER2-positive tumors seem to derive similar benefits from first-line trastuzumab compared with initially HER2-positive patients. Other series have confirmed the high concordance rate between HER2-status assessment on primary tumor and matched metastases [166].

Some preliminary reports have been published on the possible role of liquid biopsy for HER2 evaluation on circulating tumor DNA [167]. As in other malignancies, it is possible that this technique will help in monitoring treatment response during anti-HER2 therapy rather than substitute tissue assessment for HER2 evaluation.

Advanced gastric cancer comprises different clinical situations. Yoshida et al. classified stage IV disease into four subgroups based on sites involved and extent of involvement [168]. In their opinion, this could guide conversion surgery in patients benefiting from chemotherapy. Historically, two main subsets of patients have been defined: those with locally advanced unresectable disease (mainly due to primary tumor extension and/or abdominal nodal involvement) and those with distant metastatic disease [169]. Although often combined and treated as a single entity, the prognosis differs, and different approaches can be considered in these two groups. Although first-line chemotherapy is the mainstay of treatment in both cases, more intensive triplet schedule may be justified in patients with locally advanced disease, where conversion to respectability is a reasonable goal of treatment [170]. Recently, surgical strategies have been extended in selected gastric cancer patients beyond nodal involvement, to pulmonary metastases [171], liver metastases [172], and even peritoneal involvement [173]. FLOT-3 explored the role of FLOT in different patient subsets, such as those with oligometastatic disease reconsidered for surgery after response [174]. Among these patients effectively submitted to surgery, interesting OS results have been reported, thus supporting the ongoing randomized trial conducted by the German AIO Group RENAISSANCE/FLOT5 (NCT02578368).

##### Second-Line Therapy

**Unanswered questions**: Regarding patient characteristics, which are the main drivers in second-line treatment decision? Is paclitaxel plus ramucirumab the standard second-line therapy after docetaxel-based first-line or (neo-)adjuvant therapy?

##### Statements

(1) Considering the aggressiveness of the disease, often associated with rapid deterioration, many patients do not receive second-line therapy (in Western countries, ~30–40% of patients receive salvage therapies).
(2) To increase the number of patients who are candidates for second-line therapies, accurate patient monitoring during first-line therapy is mandatory, to capture early signs of clinical progression which may anticipate radiographic progression.
(3) Imaging for disease assessment should ideally be performed every 2 months in patients with metastatic disease and should be accompanied by detailed and close monitoring of clinical conditions.
(4) All patients with ECOG PS 0 or 1 should be offered second-line treatment after progression to first-line therapy.
(5) ECOG PS ≥ 2 patients are candidates for BSC alone.
(6) For patients with gastric or GEJ cancer that has progressed after first-line therapy not including a taxane, the combination of paclitaxel and ramucirumab is the preferred choice.
(7) For patients with gastric or GEJ cancer that has progressed after first-line therapy with a taxane, the combination of paclitaxel and ramucirumab should be considered in patients with previous response to first-line therapy. In all other patients, including those with contraindications to this combination, alternative options can be considered, such as single-agent ramucirumab or irinotecan.

##### Sources of Evidence

Approximately 35–40% of Western patients and up to 75% of Eastern patients are treated with second-line therapies [153]. Randomized studies and meta-analyses confirm that active treatment improves OS and QoL compared with BSC alone [158,175]. Currently, second-line treatment options do not differ according to HER2 status—recent trials did not suggest any benefit from trastuzumab beyond progression in HER2-positive gastric cancer treated with trastuzumab in first line [176]. Among cytotoxic agents, irinotecan, docetaxel, and paclitaxel are all effective agents, supported by randomized trials. In particular, the COUGAR-2 study demonstrated an OS benefit for docetaxel over BSC alone [177], whereas irinotecan (initially found effective over BSC in a small randomized trial [178]) was proved equally effective to paclitaxel (with paclitaxel associated with a more favorable safety profile) in WJOG 4007 [179] and KSCG ST10-01 [180].

Among biologic agents, only the anti-VEGFR2 antibody ramucirumab demonstrated prolonged OS compared with BSC as single-agent [159] or when tested in combination with paclitaxel against paclitaxel alone [157]. Real-life data confirmed the efficacy of ramucirumab in routine practice [181].

No validated predictive parameters exist to definitively identify patients who will benefit most from palliative chemotherapy or ramucirumab [182]. Therefore, clinical selection remains essential to maximize the benefit of salvage therapies while minimizing the risk for futile toxicity. Several clinical variables are associated with OS in second line. PS is one of the main determinants of patient outcome in most series (ECOG PS ≥ 2 being almost invariably associated with dismal prognosis and representing an exclusion criterion for randomized trials) and should therefore guide treatment allocation [183,184]. Other parameters can also be used, such as benefit (for PFS) from first-line, neutrophil-to-lymphocyte ratio, lactate dehydrogenase values, and hemoglobin levels [185]. However, external validation for these variables is lacking and they cannot be currently used to deny active treatment in individual patients but may be useful in better stratifying patient prognosis after first progression [186]. Notably, age per se does not influence the efficacy of ramucirumab and chemotherapy in second line [183,187].

A second-line therapy should be offered to all patients with preserved general conditions (ECOG PS 0–1) after progression to first-line [156]. As for initial therapy, patient comorbidities as well as residual toxicities and benefit from previous systemic treatments could inform the choice of optimal agent or regimen to be used in second line. Paclitaxel plus ramucirumab is the only combination to demonstrate an OS advantage over single-agent chemotherapy in a randomized study [157]. Therefore, it is now regarded as standard of care in fit patients in second line, whereas monotherapy with docetaxel, paclitaxel, irinotecan, or ramucirumab is generally reserved for patients judged unsuitable for combination treatment [154,155,156]. For patients benefiting from first-line therapy in terms of response and PFS (progressing ≥3–4 months after first-line therapy), retreatment with first-line agents is also an option, but cumulative toxicity should be closely monitored before and during treatment and the possibility of offering salvage agents in case of rapid progression carefully evaluated in single cases. BSC remains preferable in patients with deteriorated conditions (ECOG PS ≥ 2) [154,155,156].

In RAINBOW, patients already treated with a taxane were excluded [157]. This makes it difficult to draw conclusions on the efficacy of paclitaxel plus ramucirumab after a first-line triplet regimen such as FLOT (or in patients with early progression after adjuvant FLOT). In RAMIRIS (AIO-STO-0415), a randomized Phase 2 study investigating the role of FOLFIRI plus ramucirumab compared with paclitaxel plus ramucirumab in second line, among docetaxel-pretreated patients, PFS with FOLFIRI was superior to paclitaxel when combined with ramucirumab, supporting the ongoing Phase 3 trial [188,189]. However, taking into account licensed indications for each country, the combination of paclitaxel plus ramucirumab should also be considered for docetaxel-pretreated patients with previous response to taxane-based therapy.

**Figure 5:**
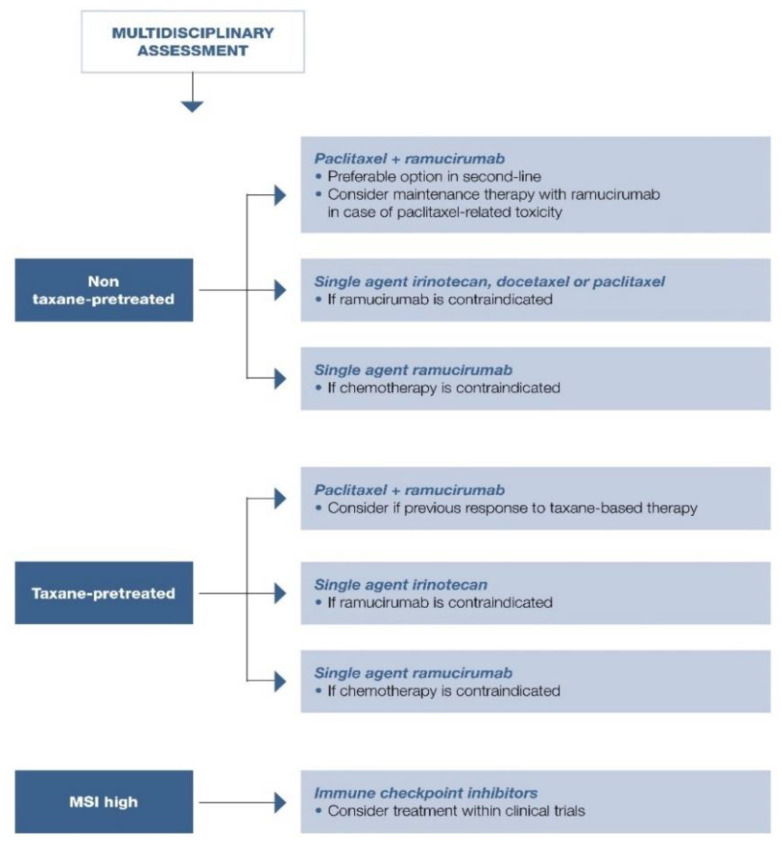
Second-line treatment options for fit patients with metastatic or unresectable gastric or gastroesophageal junction adenocarcinoma. MSI, microsatellite instability.

Second-line treatment alternatives are summarized in Figure 5. 

#### 3.6.3. Role of Maintenance Therapy After First- and Second-Line Treatment

**Unanswered questions**: What is the optimal duration of first- and second-line treatment? Is there a role for maintenance therapy? Which is the optimal maintenance therapy? Does HER2 status impact maintenance therapy selection?

##### Statements

1. Optimal treatment duration in first or second line cannot be informed by literature evidence and should be tailored to individual patient preference and tolerance.
2. The choice of shifting to maintenance treatment should be individualized after discussion with the patient about the risk-to-benefit ratio of this approach.
3. After 6 months of first-line combination treatment without evidence of progression, patients with HER2-positive disease can be offered maintenance with trastuzumab, either as single-agent or combined with fluoropyrimidine.
4. Maintenance with ramucirumab monotherapy can be considered in patients treated with second-line paclitaxel plus ramucirumab with unacceptable toxicities related to paclitaxel.

##### Sources of Evidence

No clear indication is yet available from the literature about the optimal treatment duration in first or second line. Randomized trials generally continued treatment until disease progression.

Maintenance therapy is generally offered in advanced cancer cases to prolong TTP, while preserving QoL in patients already treated with combination therapy upfront [190]. In metastatic gastroesophageal cancer, the role of such an approach is still controversial. Single-agent fluoropyrimidines have been most extensively investigated for this use; in particular, maintenance capecitabine after six cycles of XELOX prolonged PFS compared with observation alone [191]. Several smaller, uncontrolled studies confirmed these findings [192,193].

Based on data available for first-line chemotherapy (showing median PFS results for doublet or triplet rarely exceeding 6 months) and the toxicity profile of the agents most used in first-line (with the risks of cumulative toxicity for platinum compounds or taxanes), patients achieving disease control after 6 months of combination therapy are candidates for fluoropyrimidine maintenance until unacceptable toxicity, evidence of disease progression, or patient refusal [191,192,193] (Figure 4).

No evidence exists on the role of maintenance for second-line therapy. RAINBOW showed that ramucirumab can be maintained until unacceptable toxicity, evidence of disease progression, or patient refusal due to paclitaxel-related toxicities [157].

In patients with HER2-positive disease receiving combination chemotherapy plus trastuzumab, the anti-HER2 antibody should be maintained, with or without fluoropyrimidine, and continued until progression if accepted and tolerated, as scheduled in ToGA [46]. This strategy has proved well-tolerated and is accepted as the best approach to maximize the benefit of induction first-line therapy.

In HER2-negative disease, fluoropyrimidines represent the only maintenance therapy option. Ongoing randomized trials such as ARMANI [194] and MATEO [195] will clarify the role of different maintenance approaches (paclitaxel plus ramucirumab and S1, respectively) after a scheduled period of induction doublet chemotherapy.

#### 3.6.4. Role of Third-Line Therapy

**Unanswered questions**: Should a third-line treatment be offered in routine practice?

##### Statements

1. Fewer than 20% of Western gastric cancer patients are offered third-line therapies in clinical practice.
2. Patients with adequate general health (ECOG PS 0–1) who experience progression on second-line therapy may be considered for third-line therapies. Among cytotoxic agents, TAS102 (if available) is a preferred option; irinotecan (if not used in previous lines) may be considered if TAS102 is not available.

##### Sources of Evidence

Randomized trials suggest that 10–20% of patients treated for advanced disease receive third-line systemic therapy [153]. This percentage may be higher in Eastern countries, where 30–40% of selected patients included in randomized trials receive salvage therapies beyond second line [153]. Recent real-world data confirm these percentages and provide evidence of a modest benefit from cytotoxic agents not used in earlier lines [196,197].

Recently, TAS102 has been compared with placebo after two lines of therapy in a randomized Phase 3 trial [198]. TAS102 was significantly superior to placebo in terms of OS and reported a trend towards reducing the risk of QoL deterioration compared with placebo [199].

#### 3.6.5. Role of Immunotherapy in Advanced Disease

**Unanswered questions:** Is there a role for ICIs in metastatic gastroesophageal cancer? Is there any predictive biomarker for ICIs in metastatic gastroesophageal cancer? What is the cost-to-benefit ratio of ICIs?

##### Statements

1. For patients with MSI-high disease, treatment with immune checkpoint inhibitors (ICIs) should be considered if not administered in earlier lines.
2. Based on already published or presented Phase 3 studies investigating ICIs either alone or in combination with chemotherapy, ICIs should not be offered in routine practice to unselected metastatic gastric cancer patients outside clinical trials.
3. Based on available efficacy data of ICIs in MSI-high gastric cancer patients, treatment with ICIs should always be considered in this subgroup within clinical trials or as off-label use.
4. No pharmacoeconomic analyses have been published on the use of ICIs in advanced disease.

##### Sources of Evidence

Several trials have evaluated ICIs in metastatic gastroesophageal cancer. As first-line therapy, KEYNOTE-062 compared chemotherapy alone (cisplatin plus 5-FU or capecitabine) with chemotherapy plus pembrolizumab or pembrolizumab alone among patients with HER2-negative, PD-L1 CPS-positive disease [54]. The primary endpoints were OS in CPS ≥1 and ≥10 for pembrolizumab plus chemotherapy versus chemotherapy and pembrolizumab versus chemotherapy, as well as PFS in CPS ≥1 for pembrolizumab plus chemotherapy versus chemotherapy. Pembrolizumab monotherapy was non-inferior to chemotherapy but was associated with favorable safety. Adding pembrolizumab to chemotherapy did not significantly improve OS [54]. An exploratory analysis of >50 patients with MSI-high tumor showed superior RR, PFS, and OS in both pembrolizumab arms over chemotherapy alone, demonstrating that this subgroup may achieve particularly improved outcomes with pembrolizumab [200].

Another trial assessed avelumab versus continued chemotherapy as maintenance after 12 weeks’ induction oxaliplatin plus fluoropyrimidine therapy [201]. Avelumab failed to meet the primary endpoint of improving OS after induction, either in all randomized patients or the PD-L1+ subgroup (≥1% of tumor cells).

Among pretreated patients, three Phase 3 trials have been published. The ATTRACTION-2 study assessed nivolumab in patients treated with at least 2 lines of systemic therapy [202]. Nivolumab improved RR, PFS, and OS without unexpected toxicity [202]; the benefit was independent of PD-L1 status according to a post-hoc analysis in >39% of enrolled patients [202]. JAVELIN GASTRIC 300 evaluated avelumab as third-line therapy [203]. Compared with investigator’s choice chemotherapy, avelumab did not demonstrate any superiority in RR, PFS, and OS, even after patient stratification according to PD-L1 status. Finally, KEYNOTE-061 study compared pembrolizumab with paclitaxel as second-line therapy for patients with PD-L1 CPS ≥1% [119]. Pembrolizumab did not improve OS over paclitaxel and was associated with a shorter PFS and similar RR compared with chemotherapy.

Based on these results, no ICI is currently approved by European Medicines Agency (EMA) for metastatic esophagogastric cancer.

Factors that have been investigated most as potential predictive biomarkers for ICIs in this disease are:

PD-L1 expression assessed as either CPS or tumor-positive score: increased PD-L1 expression is generally associated with increased benefit from ICIs, namely pembrolizumab for PD-L1 CPS [54,119]. However, no external validity of this biomarker has been demonstrated with different ICIs [202,203].

MSI status: post-hoc analyses of randomized trials and Phase 2 studies confirm high activity and sustained efficacy of ICIs in this subgroup [118,204,205]. In randomized trials, investigational ICIs seem better than standard treatments in patients with MSI-high disease [119,200] (Figure 4 and Figure 5).

EBV status: EBV-positive and MSI-high patients harbor a greater immune infiltrate and higher PD-L1 expression [206]. Limited series are available, but data seem reassuring about the sensitivity of EBV-positive gastric cancer to ICIs [118,207].

No pharmacoeconomic analyses have yet been published. Moreover, conflicting data about the efficacy of different ICIs in different settings and patient populations, as well as mechanisms of reimbursement in different countries, preclude drawing definitive conclusions.

Data suggest that the toxicity profile of ICIs in gastric cancer is similar to that observed in other malignancies, and combining anti-PD-1 agents with chemotherapy is feasible [200]. QoL analysis for first-line therapy in KEYNOTE-062 showed similar outcomes for pembrolizumab compared with chemotherapy, but the toxicity profile of single-agent pembrolizumab is generally milder [208].

#### 3.6.6. Role of HIPEC and PIPAC in Advanced Disease

**Unanswered questions**: What is the role of hyperthermic intraperitoneal chemotherapy (HIPEC) and pressurized intraperitoneal aerosol chemotherapy (PIPAC), if any, in gastric cancer?

##### Statements

(1) Results for CRS plus HIPEC in gastric cancer with peritoneal involvement are limited and controversial. While confirmation in randomized trials is ongoing, this approach should be offered only within a clinical trial.
(2) There is currently insufficient evidence to recommend the use of PIPAC outside a clinical trial. The use of this technique may be of particular interest in treating uncontrolled malignant ascites.

##### Sources of Evidence

The use of cytoreductive surgery (CRS) plus HIPEC or PIPAC in gastric cancer is confined to clinical trials [209]. Available studies are generally not controlled, populations are heterogeneous, and results for OS conflicting [210]. These limitations do not permit the identification of optimal candidates for the procedures or for a comparison with systemic treatment alone in for OS and QoL. Although these procedures are generally performed in high-volume centers, complications are frequently reported, especially with HIPEC. Ongoing randomized trials will clarify the role of intraperitoneal therapies, as current evidence suggests the potentials of HIPEC to prolong OS [211].

#### 3.6.7. Nutritional Support During Treatment of Advanced Disease

**Unanswered questions**: What is the role of home parenteral nutrition (HPN) in metastatic gastric cancer?

##### Statement

(1) HPN is a valid therapeutic option for malnourished patients with advanced gastric cancer. To reduce the risks related to HPN, this approach should be planned and managed by dedicated personnel according to validated protocols.

##### Sources of Evidence

HPN is indicated in patients with alterations in the gastrointestinal tract precluding adequate oral caloric intake (i.e., with oral intake of <60% of required for prolonged periods) [212,213]. Parenteral nutrition is effective in improving nutritional status, QoL, and, in selected patients with advanced gastric cancer, OS [214,215,216]. Malnutrition, sarcopenia, and cachexia are poor prognostic determinants in this setting [14]. However, HPN is associated with risks of glycemic decompensation and central venous catheter infections [217], and therefore dedicated personnel within the nutritional support team should be responsible for prescribing and monitoring HPN.

**Unanswered questions**: What is the preferred timing of nutritional support, upfront versus when needed?

##### Statements

(1) Nutritional status assessment must be performed before treatment initiation in all patients who are candidates for systemic therapies.
(2) Nutritional support need and programming of such support should be established by a dedicated nutrition expert.

##### Sources of Evidence

Up to 80% of patients with advanced esophagogastric cancer present with malnutrition. Sarcopenia is also a risk factor for increased toxicity of systemic chemotherapy [14,15]. Cytotoxic agents are generally dosed according to body surface area (BSA), which does not account for differences in body composition, thus possibly impacting drug exposure in patients with similar BSAs. Moreover, chemotherapy may further impact lean body mass loss, further worsening sarcopenia in some patients [218]. Therefore, assessment of nutritional status should be part of the clinical assessment on diagnosis of advanced disease, and evaluation by a nutrition expert should be offered before treatment initiation to all malnourished patients or those at risk of malnutrition.

Enteral or parenteral nutritional support in these patients is indicated to improve body weight, allow treatment administration as scheduled, and recover from gastrointestinal toxicity of treatments. In terminal phases, nutritional support appears to be less effective, and potential risks may overwhelm benefits [212].

Studies confirm that the success of nutritional support lies on the involvement of a dedicated nutrition expert in the full course of treatment [212].

#### 3.6.8. Definition of a Continuum of Care in Advanced Gastric Cancer

**Unanswered questions**: What is the impact of centralization of medical treatment in advanced gastric cancer?

##### Statement

(1) Multidisciplinary management, rather than treatment centralization in high-volume centers, represents the most effective strategy to optimize the continuum of care of medical therapies in all treatment phases.

##### Sources of Evidence

In advanced gastroesophageal cancer, the impact of the number of patients on outcome of palliative medical therapies has not been fully evaluated. A recent Dutch registry study reported a significant, if limited, advantage in OS for patients treated in high-volume centers for medical and surgical therapies [219]. However, several factors should be considered, such as more accurate selection of fit patients in referral centers, earlier and more intensive supportive treatment, and available staff resource. On the other hand, the hospital volume was not documented to have an impact on the surgical and oncological outcomes in gastric cancer patients treated in East Asia [220].

## 4. Conclusions

The GAIN working group elaborated a detailed perspective on the major open questions in the management of gastroesophageal carcinoma, from prevention and early detection, through diagnosis and staging, to treatment and subsequent follow-up. Overall, a high rate of agreement has been reached for most statements, suggesting that a shared approach could be defined in most cases, even when conclusive evidence from literature is lacking.

A potential limitation of the current project is represented by the involvement of experts from high-volume centers across Italy, as this may have reduced the divergence among the panelists. However, we tried to move in those areas of uncertainties not exhaustively covered by literature data. Therefore, the general agreement that a multidisciplinary approach is mandatory in any patient with a new diagnosis of gastric or GEJ cancer and should be encouraged in any phase after the assessment of the results of different treatment modalities, is of note. Indeed, we believe that our project can be of use to implement the organization in established teams of the specialists involved in the management of gastroesophageal cancer patients.

## Figures and Tables

**Table 1:** Follow-up of precancerous conditions.

Precancerous Conditions	Timing of Endoscopic Follow-Up	Statement
Intestinal metaplasia limited to the gastric antrum or body	Every 3 years	Endoscopic surveillance with EGDS at least every 3 years must be considered mainly for patients with familial gastric neoplasia, autoimmune gastritis, or non-eradication of H. pylori infection.
Chronic atrophic gastritis and/or diffuse intestinal metaplasia.	Every 1–2 years	Scheduled endoscopic follow-up with controls 1–2 years is recommended.
Gastric dysplasia	Within 6 months for high-grade dysplasia and within 1 year for low-grade dysplasia.	In patients with dysplasia without suspicious lesions, first, an endoscopic evaluation at a third-level center should be performed, and possibly a review by expert pathologists to confirm the previous diagnosis and guide endoscopic follow-up. In the absence of evident lesions, even at the subsequent endoscopic evaluation, a new EGDS must be performed within 6 months for high-grade dysplasia and within 1 year for low-grade dysplasia.

EGDS, esophagogastroduodenoscopy.

**Table 2:** Multimodal approach in assessment of response during treatment.

Exam	Timing	Statement
CT scan	Diagnosis	CT scan is essential in gastric cancer staging
Echoendoscopy	Diagnosis	EUS is the most accurate staging method for definition of T parameter; its execution is therefore recommended when this impacts the treatment approach (endoscopic resection, upfront surgery, perioperative treatment).
FDG PET/CT	Diagnosis	Consider FDG PET/CT with ^18^F-FDG in addition to radiologic imaging in patients with locally advanced gastric cancer when there is no evidence of metastatic disease and in case of suspected secondary lesions at CT scan.
CT scan	Response to neoadjuvant treatment	CT scan is the gold standard in evaluating the response after neoadjuvant chemotherapy.
Echoendoscopy	Response to neoadjuvant treatment	The usefulness of EUS in evaluating the response after neoadjuvant chemotherapy or chemoradiotherapy appears limited and so it should not be used as restaging method outside clinical trials.
FDG PET/CT	Response to neoadjuvant treatment	FDG PET/CT during treatment or after the end of treatment should be useful to identify patients without any benefit from neoadjuvant treatment; however, this role is marginal outside clinical trials.
CT scan	Metastatic	Imaging for disease assessment should be preferentially performed every 2 months with metastatic disease and should be accompanied by detailed and close monitoring of patient clinical conditions.

EUS, endoscopic ultrasound; FDG PET/CT, fluorodeoxyglucose positron emission tomography/computed tomography.

**Table 3:** Nutritional support: how and when?

Nutritional Action	Stage of Disease	Statement
Role of nutrition experts	All	The presence of a clinical nutrition specialist in the MDT allows the possibility of performing multimodal treatments. The nutrition team should include at least one physician (internist, gastroenterologist, endocrinologist, or nutritionist) and a dietitian.
Nutritional screening	All	Validated screening test to assess the presence of malnutrition should be provided to all patients with gastric cancer before any anticancer treatment. For pathologic screening, the nutritional team must be involved in setting up an intervention.
Nutritional counseling	All	For pathologic nutritional screening, nutritional counseling should be the first intervention. Nutritional counseling aims to maintain adequate weight and QoL during chemotherapy.
Enteral nutrition	Localized disease	Enteral nutrition support in patients treated with radical gastrectomy should be considered in (neo-)adjuvant settings.
Parenteral nutrition	Metastatic	Parenteral nutrition is effective in improving nutritional status and QoL, and in some selected patients with advanced gastric cancer. It is a valid therapeutic option for malnourished patients with advanced gastric cancer.
Nutritional status assessment	Metastatic	Nutritional status assessment must be performed upfront in all patients who are candidates for systemic therapies. The need for nutritional support and the program for such support should be established by a dedicated nutrition expert.

QoL, quality of life.

## Data Availability

Not applicable.
